# Septic shock is correlated with asymmetrical dimethyl arginine levels, which may be influenced by a polymorphism in the dimethylarginine dimethylaminohydrolase II gene: a prospective observational study

**DOI:** 10.1186/cc5053

**Published:** 2006-09-26

**Authors:** Michael J O'Dwyer, Felicity Dempsey, Vivion Crowley, Dermot P Kelleher, Ross McManus, Thomas Ryan

**Affiliations:** 1 Department of Anaesthesia, St James's Hospital, James's St, Dublin, D7, Ireland; 2 Department of Clinical Medicine, Trinity College, Dublin, D2, Ireland; 3 Department of Clinical Chemistry, St James's Hospital, James's St, Dublin, D7, Ireland

## Abstract

**Introduction:**

Asymmetrical dimethyl arginine (ADMA) is an endogenous non-selective inhibitor of nitric oxide synthase that may influence the severity of organ failure and the occurrence of shock secondary to an infectious insult. Levels may be genetically determined by a promoter polymorphism in a regulatory gene encoding dimethylarginine dimethylaminohydrolase II (DDAH II), which functions by metabolising ADMA to citrulline. The aim of this study was to examine the association between ADMA levels and the severity of organ failure and shock in severe sepsis and also to assess the influence of a promoter polymorphism in *DDAH II *on ADMA levels.

**Methods:**

A prospective observational study was designed, and 47 intensive care unit (ICU) patients with severe sepsis and 10 healthy controls were enrolled. Serum ADMA and IL-6 were assayed on admission to the ICU and seven days later. Allelic variation for a polymorphism at position -449 in the *DDAH II *gene was assessed in each patient. Clinical and demographic details were also collected.

**Results:**

On day 1 more ADMA was detectable in the ICU group than in the control group (*p *= 0.005). Levels subsequently increased during the first week in ICU (*p *= 0.001). ADMA levels were associated with vasopressor requirements on day one (*p *= 0.001). ADMA levels and Sequential Organ Failure Assessment scores were directly associated on day one (*p *= 0.0001) and day seven (*p *= 0.002). The degree of acidaemia and lactaemia was directly correlated with ADMA levels at both time points (*p *< 0.01). On day seven, IL-6 was directly correlated with ADMA levels (*p *= 0.006). The variant allele with G at position -449 in the *DDAH II *gene was associated with increased ADMA concentrations at both time points (*p *< 0.05).

**Conclusion:**

Severity of organ failure, inflammation and presence of early shock in severe sepsis are associated with increased ADMA levels. ADMA concentrations may be influenced by a polymorphism in the *DDAH II *gene.

## Introduction

Overwhelming infection with resultant multiple organ failure, which has been termed the 'sepsis syndrome' [1], is a devastating illness, and a common intensive care unit (ICU) admission diagnosis, with an incidence of 3 per 1,000 population per annum [2]. The sepsis syndrome has been characterised as a dysregulation of inflammation in response to infection, with life-threatening organ failure attributable to a combination of excessive inflammation, disseminated coagulopathy and disruption of the integrity of microvascular endothelium [3].

Endothelium-derived nitric oxide (NO) is a potent vasodilator that antagonises the effects of endogenous vasopressors [4]. NO is produced from L-arginine by an enzyme, nitric oxide synthase (NOS), which exists in constitutive, inducible, endothelial and neuronal isoforms. The endothelial isoform (eNOS) regulates vascular tone and interactions between leukocytes and endothelium [5]. Consequently, NO has been implicated in the pathogenesis of the hypotension and organ failure attributable to severe sepsis [6]. However, although non-selective pharmacological inhibition of NOS briefly attenuates the haemodynamic anomalies seen in these patients with severe sepsis, the overall effect of such inhibition is to increase mortality [7].

This conundrum may be explained in part either by selective inhibition of the various isoforms of NOS or by an ancillary non-vascular function of NOS. Specifically, inhibition of the constitutively expressed isoform of NOS, which is essential to maintain organ perfusion, may be detrimental [8]. However, and of considerably greater importance in the context of sepsis, NO has an ancillary yet critical protective function, possessing potent antimicrobial properties, antagonism of which may account for the excess mortality observed with NOS inhibition in patients with sepsis [9].

Asymmetrical dimethyl arginine (ADMA) is a naturally occurring non-selective inhibitor of NOS, derived from protein catabolism, and is metabolised to citrulline by dimethylarginine dimethylaminohydrolase (DDAH) [10]. The co-localisation of DDAH and NOS at several sites supports the hypothesis that DDAH may regulate NOS activity by controlling the metabolism of ADMA [10]. DDAH exists as two distinct isoforms, with DDAH I present in tissues expressing neuronal *NOS*, whereas *DDAH II *has an expression pattern similar to that of eNOS [11], thus making DDAH II characteristic of vascular tissue such as the heart and endothelium. Variation in *DDAH II *expression or activity might therefore be an important mechanism in the haemodynamic alterations and end-organ damage observed in sepsis. Notably, DDAH displays decreased activity when operating in an inflammatory milieu [12]. Depletion of NO by ADMA has biological significance, because elevated ADMA levels are seen in patients with vascular disease, hepatic failure and renal failure, and are linked with greater severity of organ failure in ICU patients with sepsis [5,13]. Furthermore, it has recently been postulated that the beneficial effects of the administration of exogenous insulin may be associated with fluctuations in ADMA levels in patients with sepsis [13]. However, variation in ADMA levels may also have a genetic basis. Gene polymorphism, observed in the promoter region of the *DDAH II *gene, may have functional significance [14] but has not previously been studied in a human population with sepsis. However, an association between gene polymorphism in the promoter region of the *DDAH II *gene and systemic arterial vasodilation after cardiac surgery with cardiopulmonary bypass suggests a link between pathological vasodilation, such as that occurging with severe sepsis, and ADMA metabolism [15].

We undertook a study to assess the relationship between ADMA levels and organ failure in ICU patients with severe sepsis and also to assess the possible functionality of a polymorphism in the *DDAH II *promoter, designated *DDAH II *-449 (single-nucleotide polymorphism (SNP) ID rs805305).

## Materials and methods

This study was conducted in the ICU of St James's Hospital, Dublin, Ireland, and was approved by the local research ethics committee. Informed written consent was obtained from each patient or a first-degree relative. A total of 47 consecutive patients with severe sepsis or septic shock, as defined by the American College of Chest Physicians/Society of Critical Care Medicine Consensus Conference [1] were enrolled. Ten healthy staff members served as a control group.

Severity of illness was characterised with the Sequential Organ Failure Assessment (SOFA) scoring system [16] and the Simplified Acute Physiology Score (SAPS2) [17] on admission to ICU, and with the SOFA score again on day seven. Individual clinical and laboratory variables relating to inflammation were collected on days one and seven of ICU stay. The recorded variables represented the most significant derangements from normal values recorded over each 24-hour period. The requirement for vasoactive or vasopressor medications to maintain a mean arterial pressure greater than 60 mmHg was recorded. These medications consisted of either adrenaline or noradrenaline infusions. Death in ICU or survival to ICU discharge was recorded.

Blood sampling was performed within the first 24 hours of ICU admission and again seven days later through an indwelling central venous line. Serum was obtained from whole blood clotted for 30 minutes at room temperature and spun at 2,500 rev./minute for 10 minutes.

ADMA was measured with a microtitre plate assay (DLD Diagnostika Ltd, Hamburg, Germany) as described previously [18].

Serum IL-6 concentrations were measured by ELISA (R&D Systems, Minneapolis, MN, USA) in accordance with the manufacturer's instructions. The lower limit of detection for IL-6 was 9.4 pg/ml. All samples were tested in duplicate.

Genomic DNA was extracted from whole blood with a commercially available DNA isolation kit (QIAmp DNA blood Midi kit, Qiagen GmBH, Crawley, West Sussex, UK). Allelic variation for the polymorphism was assayed using Amplifluor technology by Kbiosciences (Hoddesdon, Herts., UK). Primer sequences are listed in Table 1.

Statistical analysis was performed with the JMP software package (SAS, Cary, NC, USA). Between-group comparisons for continuous variables were analysed by Wilcoxon rank sum test, Wilcoxon sign rank test and Kruskal-Wallis test where appropriate. Spearman's rank correlation coefficient was used to analyse the relationship between continuous variables. For all comparisons, *p *< 0.05 was considered significant.

## Results

Consent was gained for 47 ICU patients and from 10 healthy controls; they were recruited into the study. Blood samples were available for analysis from 40 patients on day 1 and from 35 patients on day 7; 28 patients had blood samples available for analysis at both time points. Fourteen (30%) patients died before discharge from ICU. Demographic data, clinical details and levels of inflammatory markers for patients are detailed in Tables 2 to 4.

### Day one comparisons

On day one, 31 patients (66%) required infusion of a vasoactive compound to maintain adequate arterial pressure. ADMA levels (*p *= 0.001), lactate levels (*p *= 0.018) and organ failure scores (*p *< 0.003) were higher in this group requiring vasoactive infusions (Table 3). Patients in this group on day one were also more likely to be non-survivors (*p *= 0.01; Table 3).

Plasma lactate levels were directly correlated with ADMA levels on day 1 (*r*^2 ^= 0.28, *n *= 40, *p *= 0.0003). In addition, SOFA score and ADMA levels were directly correlated on day 1 (*r*^2 ^= 0.31, *n *= 40, *p *< 0.0001).

To elucidate whether the relationship between ADMA levels and SOFA score was entirely attributable to cardiovascular failure, a non-cardiac organ failure score was obtained by excluding the cardiovascular component from the total SOFA score. There was a positive correlation between this score and ADMA levels on day 1 (*r*^2 ^= 0.23, *n *= 40, *p *= 0.002).

ADMA levels on day 1 were not related to survival, nor were the highest producers of ADMA (highest quartile) more likely to have a higher mortality. However, SOFA scores and IL-6 levels on day one did distinguish between survivors and non-survivors on day 1 (*p *= 0.02 and *p *= 0.001, respectively) (Table 2).

### Day seven comparisons

On day seven, 10 patients (24%) required infusion with vasoactive medication to maintain a normal blood pressure. Although there was a trend towards increasing ADMA levels in those patients requiring vasoactive infusions to maintain blood pressure, this did not reach significance (*p *= 0.07; Table 4).

Plasma lactate levels were directly correlated with ADMA levels on day 7 (*r*^2 ^= 0.18, *n *= 31, *p *= 0.01). In addition, SOFA score and ADMA levels were directly correlated on day 7 (*r*^2 ^= 0.23, *n *= 35, *p *= 0.002). The non-cardiac organ failure score was calculated as above from the day 7 SOFA score. This score was positively correlated with ADMA levels on day 7 (*r*^2 ^= 0.22, *n *= 35, *p *= 0.005).

ADMA levels on day 7 were not related to survival, nor were the highest producers of ADMA (highest quartile) more likely to have a higher mortality. However, increased SOFA scores, acidosis and requirement for infusion of vasoactive medications on day 7 were associated with increased risk of death (Table 2).

### ADMA levels by group

On the first day of critical illness, the ICU group had greater ADMA levels than the control group (*p *= 0.005). ADMA levels subsequently rose over the first week in the ICU group (*p *= 0.001; Table 5).

### Correlation between ADMA levels and inflammatory markers

Various inflammatory markers were correlated with ADMA levels on univariate analysis on both day 1 and day 7 (Table 6). Whereas pH, base excess and lactate levels were correlated with ADMA on both day 1 and day 7, IL-6 levels were correlated with ADMA only on day 7, and the white cell count was not correlated with ADMA at either time point (Table 6).

### Correlation between severity of organ failure and ADMA and IL-6 levels

Multivariate analysis of the relationship between the SOFA scores and the biological markers ADMA and IL-6 revealed that on day 1 both ADMA (*p *= 0.002) and IL-6 (*p *= 0.009) were independently related to SOFA scores, whereas on day 7 only ADMA (*p *= 0.002) was independently related to the SOFA score (Table 7).

### Allelic variations

The distribution of *DDAH II *alleles conformed to a Hardy-Weinberg equilibrium. There was no association between any clinical outcome measure and carriage of specific *DDAH II *alleles. Twenty-four patients (45%) were GG homozygotes, 5 (11%) were CC homozygotes and 19 (43%) were heterozygotes at position -449 in the *DDAH II *promoter. There was a trend towards increasing amounts of ADMA between different *DDAH II *genotypes. ADMA was most abundant in the GG homozygotes, least abundant in the CC homozygotes and detectable at intermediate levels in the heterozygotes. This trend was present at both time points, although it failed to reach significance on either day 1 (*p *= 0.069) or on day 7 (*p *= 0.32). However, carriage of the G allele at position -449 was associated with increased ADMA production on both day 1 (*p *= 0.03) and day 7 (*p *= 0.042) (Table 8).

## Discussion

There are limited data on the role of ADMA and DDAH II in systemic inflammation, with two studies of critically ill patients observing a relationship between the highest producers of ADMA and fatal outcome [5,13]. Although our study may not have been adequately powered to detect outcome variations, we have demonstrated both an increase in ADMA levels in critically ill patients in comparison with healthy controls and described an association between increasing ADMA levels, the occurrence of septic shock and greater severity of organ failure.

Given the ubiquitous involvement of NO in vascular regulation and leukocyte function, the consequences of excess ADMA in inflammatory and septic states are likely to be manifold. Raised ADMA levels may lead to pathogenic changes in the microvasculature by inhibiting constitutively expressed NOS [8]. The consequent loss of basal NO production may lead to impaired blood flow with platelet aggregation, causing endothelial damage, interstitial oedema and resultant organ failure [19].

However, ADMA mediated inhibition of inducible NOS (iNOS) in patients with sepsis may interfere with macrophage bactericidal properties, because NO is an essential component in the phagocytic response to bacterial infection. Interferon-γ, released in response to an infective insult, acts on macrophages to increase the expression of iNOS [9]. This activates the cells to a heightened microbicidal state, mediated by NO and adducts of the nitrogenous products of nitric oxide synthases. As a consequence mice with a non-functional *iNOS *gene are susceptible to infection [20]. Furthermore, in clinical trials of NOS inhibition in patients with sepsis, although NOS inhibition ameliorates pathogenic vasodilation and lessens vasopressor requirement, the overall effect is to compromise survival [7]. This suggests, in the context of severe sepsis, that NO-linked immune mechanisms are of greater importance than NO-meditated vascular regulation.

We observed that elevated ADMA levels are correlated with vasopressor support in early septic shock. Although this may seem counterintuitive because previous evidence implicated NO in the pathogenesis of the hypotension observed in septic shock [21], it is plausible that inappropriately increased ADMA levels may impair macrophage function by means of NOS inhibition. The associated inflammatory response to an unresolved infection may be partly responsible for the observed hypotension and organ failure operating through an alternative mechanism. This persistent inflammatory response is reflected in the linkages between IL-6, ADMA and the severity of organ failure (Tables 6 and 7). The association with IL-6 is noteworthy because this is a well-recognised marker of generalised inflammation, consistently elevated in patients with sepsis [22].

About 90% of ADMA is metabolised by the enzyme DDAH [10]. It is possible that variation in ADMA levels in patients with sepsis is reactive and represents an epiphenomenon. However, we observed that carriage of a G at position -449 in the promoter region of the *DDAH II *gene is associated with increased ADMA levels, which suggests that the *DDAH II *gene with a G at this position is less active than that with a C. The more active isoform results in lower ADMA levels, less iNOS inhibition and consequently an appropriate bactericidal phagocytic response. It is noteworthy that *DDAH II *maps to 6p21.3, a region of DNA that is particularly rich in genes involved in immune and inflammatory responses. It has been hypothesised that this location and wide expression in immune cells make it a candidate as a disease susceptibility gene in sepsis [10].

We have previously described an association between the presence of a G at position -449 in the *DDAH II *gene and the requirement for vasopressors after cardiopulmonary bypass during cardiac surgery [15]. Although this is the opposite of what we observed in septic patients, it is noteworthy that the two insults are also quite different. The cardiopulmonary bypass circuit invokes a sterile inflammatory response, whereas the ICU patients with sepsis received an infective inflammatory insult. Consequently, the role of ADMA in manipulating NO levels may be context sensitive. NO may have pivotal beneficial bactericidal properties necessary for the resolution of a septic insult while contributing to an undesirable vasodilatory state in the setting of a sterile inflammatory insult.

This potential genetic component to the fluctuations observed in ADMA levels secondary to a septic insult may help to explain some of the residual variability observed in a previous study attempting to link exogenous insulin administration to ADMA levels [13]. Thus, interindividual variability in ADMA production is likely to be multifactorial, with contributions from genetic and environmental factors.

## Conclusion

We have confirmed the association between ADMA levels and the extent of multiple organ failure in sepsis. We have also demonstrated that ADMA levels are upregulated in response to an infective insult and are also associated with hypotension in this setting. We hypothesise that this may be due to ineffective bactericidal activity of macrophages and persistent inflammation. Finally, we suggest that ADMA levels may be regulated via a genetic component. We propose that a polymorphism at position -449 in the *DDAH II *may be functional and has the potential to be used as a marker for the susceptibility to and severity of an inflammatory response secondary to an infective insult. A larger study will be required to confirm these findings.

## Key messages

• ADMA, an endogenous non-selective inhibitor of NOS, may have a key role in vascular regulation.

• Compromised NO production may influence morbidity by disrupting microcirculatory blood flow and also could potentially compromise key bactericidal functions in the host.

• Increased ADMA levels are associated with multiple organ failure and shock in the setting of a septic insult.

• ADMA may be regulated by means of host genetic mechanisms, which influence the efficiency of the enzymatic breakdown of ADMA by *DDAH II*.

## Abbreviations

ADMA = asymmetrical dimethyl arginine; DDAH = dimethylarginine dimethylaminohydrolase; ELISA = enzyme-linked immunosorbent assay; eNOS = endothelial NO synthase; iNOS = inducible NO synthase; ICU = intensive care unit; IL = interleukin; NO = nitric oxide; NOS = nitric oxide synthase; SOFA = Sequential Organ Failure Assessment.

## Competing interests

The authors declare that they have no competing interests.

## Authors' contributions

MO'D participated in the design of the study, patient recruitment, data and sample collection, ELISA and DNA analysis, statistical analysis, and drafting of the manuscript. FD and VC participated in the ADMA analysis. DK participated in the design of the study and drafting of the manuscript. RM participated in the design of the study, genotype analysis, statistical analysis and drafting of the manuscript. TR participated in the design of the study, patient recruitment, statistical analysis and drafting of the manuscript. All authors read and approved the final manuscript.

## Figures and Tables

**Table 1:** 

Locus		Primer
DDAHII-449	Allele 1	GAAGGTGACCAAGTTCATGCTGACTGGAAGTCCAGCCCGG
	Allele 2	GAAGGTCGGAGTCAACGGATTGACTGGAAGTCCAGCCCGC
	Common	CCAGCTTTCTCCTTCTGTCCCATAA

**Table 2:** Demographics and asymmetrical dimethyl arginine (ADMA) levels by group

Parameter	Survivors	Non-survivors	*p*
Total patients	33 (70)	14 (30)	
Male sex	17 (52)	9 (64)	ns
SOFA score, day 1	7 (4–10)	9 (8.75–12.5)	0.02
SOFA score, day 7	4 (2.75–7.25)	10.5 (8.5–11.75)	0.006
SAPS2 score	39 (30.5–51.5)	47.5 (39.5–60.75)	ns
Lactate, day 1	2.1 (1.5–5)	3.6 (2.25–5.75)	ns
Lactate, day 7	1.5 (1–1.9)	2 (1.1–4)	ns
Vasoactive agents, day 1	18 (55)	13 (93)	0.01
Vasoactive agents, day 7	5 (15)	5 (63)	0.005
ADMA, day 1	0.88 (0.52–1.09)	0.91 (0.64–1.23)	ns
ADMA, day 7	1.05 (0.66–1.21)	1.24 (0.77–1.53)	ns
pH, day 1	7.31 (7.26–7.38)	7.33 (7.27–7.37)	ns
pH, day 7	7.44 (7.40–7.45)	7.32 (7.24–7.40)	0.002
WCC, day 1	18 (10.7–23)	9.1 (2.3–17.8)	ns
WCC, day 7	11.6 (8.6–17.4)	13.9 (8.5–19.3)	ns
Base excess, day 1	-2.9 (-6.45 to 1.75)	-4.1 (-9.3 to 1.75)	ns
Base excess, day 7	2.9 (1.1–5.6)	0.1 (-3 to 1.33)	0.009
IL-6, day 1	277 (107–499)	782 (566–1,060)	0.001
IL-6, day 7	22 (0–651)	130 (68–425)	ns

All values are shown either as absolute counts with percentages in parenthesis or as medians with interquartile ranges in parenthesis. ADMA was measured in μmol/l, lactate in mmol/l, and IL-6 in pg/ml. SOFA, Sequential Organ Failure Assessment score; SAPS, simplified acute physiology score; ADMA, asymmetrical dimethyl arginine; WCC, white cell count; ns, not significant.

**Table 3:** Requirement for vasoactive infusions on day 1

Parameter	Vasoactive infusions	No vasoactive infusions	*p*
Total patients	31 (66)	16 (34)	
Death	13 (42)	1 (6)	0.01
IL-6	354 (189–768)	293 (87–657)	ns
pH	7.31 (7.26–7.35)	7.32 (7.29–7.40)	ns
Lactate	3.5 (1.9–6.02)	1.8 (1.05–3.75)	0.018
Base excess	-3.5 (-9 to 3.4)	-2 (-4.95 to 0.55)	ns
SOFA	9 (8–12)	4 (3.25–4.75)	<0.0001
SAPS2	47 (38–63)	33.5 (21–41.75)	0.003
ADMA	0.96 (0.82–1.29)	0.54 (0.48–0.78)	0.001
WCC	14 (8–22)	17 (8–24)	ns
MAP	65 (60–70)	79 (66–80)	0.001
Heart rate	110 (90–120)	103 (96–118)	ns
CVP	13 (10–16)	11 (7–12)	ns
Noradrenaline	13 (5–26)	-	

All values are shown either as absolute counts with percentages in parenthesis or as medians with interquartile ranges in parenthesis. IL-6 was measured in pg/ml, asymmetrical dimethyl arginine (ADMA) in μmol/l, lactate in mmol/l, and mean arterial pressure (MAP) and central venous pressure (CVP) in mmHg. Noradrenaline dosage was measured in μg/minute. SOFA, Sequential Organ Failure Assessment score; SAPS, simplified acute physiology score; WCC, white cell count; ns, not significant.

**Table 4:** Use of vasoactive infusions on day 7

Parameter	Vasoactive infusions	No vasoactive infusions	*p*
Total patients	10 (24)	31 (76)	
Death	5 (50)	3 (10)	0.005
IL-6	299 (20–784)	23 (0–117)	0.037
pH	7.36 (7.23–7.41)	7.44 (7.40–7.45)	0.0006
Lactate	2.35 (1.48–3.73)	1.2 (1–1.8)	0.006
Base excess	0.05 (4.7–3.8)	2.9 (1.1–4.8)	0.04
SOFA	11 (10–15)	4 (2.3–5.8)	<0.0001
ADMA	1.21 (0.88–1.57)	1 (0.66–1.18)	ns
WCC	18.2 (13.3–22.2)	10 (8.2–13.8)	0.01
MAP	70 (69–76)	82 (80–90)	0.01
Heart rate	100 (85–111)	78 (70–90)	0.04
CVP	11 (10–12)	10 (9–14)	ns
Noradrenaline	12 (4–21)	-	

All values are shown either as absolute counts with percentages in parenthesis or as medians with interquartile ranges in parenthesis. IL-6 was measured in pg/ml, asymmetrical dimethyl arginine (ADMA) in μmol/l, lactate in mmol/l, and mean arterial pressure (MAP) and central venous pressure (CVP) in mmHg. Noradrenaline dosage was measured in μg/minute. SOFA, Sequential Organ Failure Assessment score; WCC, white cell count; ns, not significant.

**Table 5:** Asymmetrical dimethyl arginine (ADMA) levels by group

Parameter	Control	Day 1 ICU^a^	Day 7 ICU^b^
ADMA	0.63 (0.57–0.71)	0.89 (0.57–1.09)	1.05 (0.71–1.32)

All values are in μmol/l and are presented as medians with interquartile ranges in parenthesis. ICU, intensive care unit. ^a^Comparison between day 1 ICU and control group by Wilcoxon rank sum test; *p *= 0.005. ^b^Comparison between day 1 ICU and day 7 ICU by Wilcoxon signed rank test; *p *= 0.001.

**Table 6:** Correlation matrix of asymmetrical dimethyl arginine (ADMA) and inflammatory markers

Parameter	ADMA day 1 data	ADMA day 7 data
pH	0.31 (0.0002)	0.32 (0.001)
Base excess	0.13 (0.02)	0.24 (0.005)
Lactate	0.29 (0.0003)	0.20 (0.01)
WCC	ns	ns
IL-6	ns	0.25 (0.006)

Values are *r*^2 ^with *p *values in parenthesis. WCC, white cell count; ns, not significant.

**Table 7:** Multivariate linear regression between SOFA scores and ADMA and IL-6 levels

Parameter	*F *ratio	*p*
Day 1 (*n *= 36, *r*^2 ^= 0.35)		
ADMA	11	0.002
IL-6	7.8	0.009
Day 7 (*n *= 30, *r*^2 ^= 0.32)		
ADMA	12.02	0.002
IL-6	0.88	ns

SOFA, Sequential Organ Failure Assessment score; ADMA, asymmetrical dimethyl arginine; ns, not significant.

**Table 8:** Variation in asymmetrical dimethyl arginine (ADMA) levels with carriage of specific alleles at DDAH II -449

Day	ADMA (μmol/l)		*p*
		
	GC/GG genotype	CC genotype	
1	0.91 (0.63–1.16)	0.51 (0.45–0.70)	0.03
7	1.06 (0.77–1.35)	0.835 (0.67–1.03)	0.04

Values are medians with interquartile ranges in parenthesis. Patients carrying variant allele with G at position -449 have either a GG or a GC genotype. DDAH, dimethylarginine dimethylaminohydrolase.
